# Integral Dose and Radiation-Induced Secondary Malignancies: Comparison between Stereotactic Body Radiation Therapy and Three-Dimensional Conformal Radiotherapy

**DOI:** 10.3390/ijerph9114223

**Published:** 2012-11-19

**Authors:** Marco D’Arienzo, Stefano G. Masciullo, Vitaliana de Sanctis, Mattia F. Osti, Laura Chiacchiararelli, Riccardo M. Enrici

**Affiliations:** 1 National Institute of Ionizing Radiation Metrology, ENEA Casaccia Research Center, Rome, Italy/Via Anguillarese 201, Rome 00123, Italy; 2 Radiation Oncology Department, Sant’Andrea Hospital, University of Rome La Sapienza/Via di Grottarossa, 1035-1039, Rome 00189, Italy; Email: masciullos@alice.it (S.G.M.); dsvita@libero.it (V.D.S.); mattiaosti@yahoo.it (M.F.O.); riccardo.maurizienrici@uniroma1.it (R.M.E.); 3 Medical Physics Department, Sant’Andrea Hospital, University of Rome La Sapienza/Via di Grottarossa, 1035-1039, Rome 00189, Italy; Email: laurac@ospedalesantandrea.it

**Keywords:** stereotactic body radiation therapy, integral dose, linear-quadratic model, biologically effective dose, BED, radio-induced secondary malignancies

## Abstract

The aim of the present paper is to compare the integral dose received by non-tumor tissue (NTID) in stereotactic body radiation therapy (SBRT) with modified LINAC with that received by three-dimensional conformal radiotherapy (3D-CRT), estimating possible correlations between NTID and radiation-induced secondary malignancy risk. Eight patients with intrathoracic lesions were treated with SBRT, 23 Gy × 1 fraction. All patients were then replanned for 3D-CRT, maintaining the same target coverage and applying a dose scheme of 2 Gy × 32 fractions. The dose equivalence between the different treatment modalities was achieved assuming α/β = 10Gy for tumor tissue and imposing the same biological effective dose (BED) on the target (BED = 76Gy_10_). Total NTIDs for both techniques was calculated considering α/β = 3Gy for healthy tissue. Excess absolute cancer risk (EAR) was calculated for various organs using a mechanistic model that includes fractionation effects. A paired two-tailed Student *t*-test was performed to determine statistically significant differences between the data (*p* ≤ 0.05). Our study indicates that despite the fact that for all patients integral dose is higher for SBRT treatments than 3D-CRT (*p* = 0.002), secondary cancer risk associated to SBRT patients is significantly smaller than that calculated for 3D-CRT (*p* = 0.001). This suggests that integral dose is not a good estimator for quantifying cancer induction. Indeed, for the model and parameters used, hypofractionated radiotherapy has the potential for secondary cancer reduction. The development of reliable secondary cancer risk models seems to be a key issue in fractionated radiotherapy. Further assessments of integral doses received with 3D-CRT and other special techniques are also strongly encouraged.

## Acronyms

3D-CRT: Three Dimensional Conformal Radiation Therapy
BED: Biological Effective Dose
CTV: Clinical Target Volume
DVH: Dose Volume Histogram
EAR: Excess Absolute Risk
EQID: Equivalent Integral Dose
GTV: Gross Target Volume
ID: Integral Dose
IMRT: Intensity Modulated Radiation Therapy
NTID: Normal Tissue Integral Dose
NTT: Non Tumor Tissue
OAR: Organ at Risk
OED: Organ Equivalent Dose
PTV: Planning Target Volume
RED: Risk Equivalent Dose
SBRT: Stereotactic Body Radiation Therapy
TPS: Treatment Planning System

## 1. Introduction

Radiotherapy has been often described as a “two edged sword” because, if on one hand it is a major modality of cancer treatment, but on the other it can be a cause of cancer. During the past decade, radiation-induced secondary malignancies have become a major concern and recent studies have shown that radiotherapy treatment is associated with a small, though statistically significant, enhancement in the risk of secondary cancers. At present it is generally agreed that around 10% of patients may develop a second malignancy due to radiation therapy, even if this number is not known with much certainty and could range between 6% and 13% [1,2]. One of the largest study, carried out on about 29,000 patients which received radiotherapy after surgery of breast cancer, showed an increase of cancer risk in non-affected breast from 7.5% to 9.3% after 15 years [3]. Another study performed by Brenner *et al*. [4] in more than 120,000 patients with prostate cancer showed an increased cancer risk after radiotherapy of 6% if compared to the group that underwent only surgery.

Modern radiotherapy techniques are moving in the direction of optimizing the dose conformation to tumors meanwhile sparing the exposure of organs at risk and minimizing the radiation load to healthy tissues; this is usually obtained by improving patient positioning, target localization and providing sharp dose gradients. In this context, new high-precision technologies like intensity modulated radiotherapy (IMRT) and SBRT represent major advances in cancer treatment. The high degree of conformity associated with these techniques is often obtained by increasing the number of fields and using fixed-shape or dynamic conformal arc beams. This has important implications in the debate over the possible increase of secondary cancers due to radiation therapy, essentially for two reasons. Firstly, delivery of a specified dose from these special techniques requires the accelerator to be energized for longer (more monitor units are needed) and, as a consequence, the total body dose due to leakage radiation is increased by a factor of two or three [5]. Secondly, if on the one hand these techniques lead to tight dose conformations and sharp dose gradients, on the other they are likely to increase the integral dose exposure to non-tumor tissues since larger volumes of normal tissues can be exposed to lower doses [6].

The increase of energy deposition in healthy tissues might play a leading role in the induction of secondary cancers, especially in the light of past and recent literature data which show a possible correlation between integral dose and secondary malignancies [7,8,9,10].

The aim of this work was to compare the integral dose (ID) imparted by SBRT and 3D-CRT and establish possible correlations between integral dose calculated from differential DVHs and the increase of carcinogenic risk.

The ID attempts to describe energy deposition within the whole body and it is historically considered as a physical quantity capable of representing the “physical aggression” and risk of complications due to radiation therapy. Integral dose is the product of mass of tissue irradiated and absorbed dose. Although it is generally accepted that normal tissue complication risk and secondary malignancies risk increase as the ID increases, ID is rarely used in clinical practice to compare competing plans or to evaluate treatment outcome. At present, it is still unknown which increase of integral dose could be considered clinically acceptable; however, as a general rule, it is recommended to keep ID to a minimum, tumor dose being fixed and provided that normal tissues are not unacceptably compromised [11]. Over the last decade many studies have attempted to compare integral dose received by different x-ray irradiation techniques (e.g., IMRT *vs*. 3D-CRT). In these studies ID was calculated by DVHs or by the product of mean dose and irradiated volume [12,13,14,15,16]. However, in none of these studies was an attempt made to estimate the potential consequences of integral dose increase.

To our knowledge, there are no studies investigating ID in stereotactic body radiation therapy and comparing SBRT with “traditional” techniques. Generally, SBRT does not lead to unacceptable side effects if the serial organs are excluded from high dose regions and the organs at risk constraints are respected. Most importantly, we wondered whether an increase of ID may be related to an increase in secondary cancer risk. This issue is not trivial since ID calculation does not consider fractionation effects, which are supposed to play a key role in radiation-induced malignancies.

Furthermore, the induction of secondary cancers is a matter of interest in SBRT as the use of this technique might be extended to patients who live for many years after radiotherapy. Given the very encouraging results with the SBRT technique, phase III studies are in fact strongly needed in order to compare SBRT with surgery in operable patients [17]. Lastly, considering the dose-escalation and retreatment possibilities provided by SBRT, the overall integral dose might reach levels considerably higher than achieved with conventional planning techniques and delivery schemes.

## 2. Materials and Methods

### 2.1. Integral Dose Evaluation

The integral dose ID to an organ *j* divided into *m* voxels is given by the following equation:

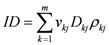
(1)


where *v_kj_*, *D_kj_* and *ρ_kj_* are respectively the volume, dose and density of voxel *k *in organ *j*. If the voxels have all the same size and the organ can be assumed to have a uniform density, Equation (1) can be reduced to:

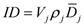
(2)
where 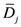 is the mean organ dose. In this study, for all treatment plans integral dose was calculated through the differential DVH using Equation (1). Although for a proper evaluation of integral dose different density values should be considered for different structures and for different bins, for the sake of simplicity a constant density *ρ* = 1 g/cm^3^ was assumed for all bins.

By changing the fractionation scheme of a certain treatment plan, the ID also changes. The basic mathematics of fractionation change are given in the Appendix. Combining Equation (2) with Equation (A-5) (Appendix), it is possible to calculate the integral dose for a given fraction regimen which is biologically equivalent to another fractionation scheme; the new integral dose to an organ *j* divided into *m* voxels (denoted with subscript 2), which is now biologically equivalent to another fractionation scheme (denoted with subscript 1), is here called EQID (Equivalent Integral Dose). Assuming that the bin size and the density remain the same, EQID is defined by the equation:

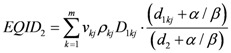
(3)
where *D_1kj_* and *d_1kj_* represents respectively the total dose and the fraction dose of voxel *k* in organ *j*, and α/β is the specific organ radiation sensitivity.

### 2.2. Dose Response Relationship for Radiation Induced Second Malignancies

There is great uncertainty regarding the dose-response relationship for induced secondary cancers in radiation therapy [18,19,20]. Currently, no accurate risk model exists and some approximations are unavoidably necessary to model data.

At present, simple models that predict risk for radiation-induced malignancies for radiation therapy are based on conventional concepts derived from radiation protection. In particular, a linear extrapolation of cancer risks from intermediate to very low doses appears to be the generally accepted methodology in ICRP [21] and BEIR [22]. In this case, most of the available information are derived from atomic bomb (A-bomb) survivor studies. These models currently provide satisfactory estimates of solid tumor risk considering a population that received a single whole body exposure. Data from A-bomb survivors show that the risk of solid tumors for total body irradiation is linear up to about 2 Gy, reaching about 8% [6,23,24].

Nevertheless, radiation protection models have to be applied with extreme care to radiotherapy patients, since doses and dose rates are quite different from those received by A-bomb survivors. In fact, A-bomb survivors received a single dose exposure of radiation, whereas radiotherapy patients receive fractionated therapy over an extended period, thus allowing for some repair of DNA damage. Further, above 1 Gy, the A-bomb survivor data are better fitted by linear-quadratic or linear-quadratic-exponential models [25].

In the past, different authors have used the dose-response relationship for A-bomb survivors to assess induced secondary cancers in radiation therapy by applying a correction dose-rate effectiveness factor (DREF) to take into account exposure to different doses and dose rates [6,26].

In the present study secondary cancer risk estimations were performed applying the mechanistic model proposed by Schneider *et al*. [27,28,29,30] for predicting cancer induction after radiotherapy, therefore including fractionation effects.

The model is based on the linear-quadratic formalism, where inductions of carcinomas and sarcomas are modeled separately and described in terms of analytical functions. The linear quadratic model of cell kill is combined with the linear-no-threshold model for radiation induced cancer at low dose in order to determine a possible dose-response relationship for radiation-induced solid cancer for radiotherapy doses.

According to this approach, for any three-dimensional inhomogeneous dose distribution, the excess absolute risk (EAR) can be calculated using the formalism described in [30] and based on the concept of organ equivalent dose, OED [28]. This model has already been successfully applied to hypofractionated radiation therapy [31]. The general properties of the model applied are discussed elsewhere [30] and will not be repeated here. However, in the following an overview of the formalism is provided.

### 2.3. Evaluation of the Excess Absolute Risk for Carcinoma Induction

According to the model proposed by Schneider *et al*. [27,28,29,30], if the dose-volume histogram *V *(*d*) of an organ or region of interest is known, the excess absolute risk in that organ, *EAR_org_*, can be calculated as follows:

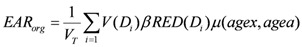
(4)
where *V_T_* is the total organ volume and β is the slope of the dose-response curve at low dose. The quantity 
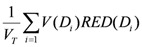
 is also known as organ equivalent dose (OED) over the whole organ volume [28,30].

The site-specific parameter β is taken from [30] and listed in Table 1 for each organ of interest in the present study. The modifying function *μ* (*agex*, *agea*) in Equation (4) contains the population dependent variables:

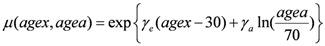
(5)


In this form the function *μ* (*agex*, *agea*) depends on the age at exposure (*agex*), the attained age (*agea*) and two organ-dependent parameters *γ_e_* and *γ_a_*, taken from [30] and listed in Table 1.

**Table 1:** Parameters used for EAR calculation according to Equations (4)–(7). For each organ, the parameters β (expressed as excess case per 10,000 PY/Gy), *γ_e_*, *γ_a_* and *R* were taken from [30]. For esophagus *R* = 0.5 was assumed since no specific *R* value is reported in [30].

Organ	Β *	*γ_e_*	*γ_a_*	α/β (Gy)	*R*	
					3D-CRT	SBRT
All Solid	74.0	−0.024	2.38	3	0.17	0
Lung	8.0	0.002	4.23	3	0.83	0
Rectum	0.73	−0.024	2.38	3	0.56	0
Esophagus	3.2	−0.002	1.9	3	0.50	0
Small Intestine	10	−0.056	6.9	3	0.09	0
Liver	2.4	−0.021	3.6	3	0.29	0
Bladder	3.8	−0.024	2.38	3	0.06	0

The risk equivalent dose, *RED *(*D*), in Equation (4) for carcinoma induction can be calculated as follows [30]:

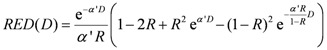
(6)
where *R* is the repopulation/repair-ability of tissues between two dose fractions. The parameter α' is defined as follows from the linear quadratic model:

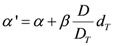
(7)
where α and β are site-specific radiosensitivity parameters, *D_T_* is the prescribed dose to the target volume, *d_T_* is the corresponding fractionation dose and *D* is the absorbed dose to the organ or region of interest.

In the limit of *R* = 0 (no repair), Equation (6) becomes:

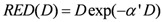
(8)


The repopulation parameter *R* in Equation (6) characterizes the repopulation/repairability of the tissue between two dose fractions. This allows two limiting cases: (1) the first one (bell-shaped dose-response curve) is obtained by neglecting any repopulation/repair effect (*R* = 0) and thus fractionation. This approach was used for EAR assessment in SBRT. (2) The second case (plateau dose response curve) is obtained considering full repopulation/repair (*R* = 1). EAR for 3D-CRT was evaluated considering *R* values ranging from 0.06 and 0.83, derived from [30] and reported in Table 1. For esophagus *R* = 0.5 was assumed since no specific *R* value is reported in [30].

### 2.4. Patient Selection and Treatment Planning

Eight patients with intrathoracic lesions were planned and treated with SBRT with a single-dose of 23 Gy. The integral doses to PTV and non tumor tissue (NTT) were then calculated. NTT was determined as “healthy tissue volume—tumor volume”, *i.e*.,

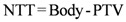
(9)


For each patient, the planning volumes were well within the planning CT scans, so that the irradiated normal tissues were included in the CT volumes.

All SBRT treatment were re-planned in standard 3D-CRT with a 2 Gy/fraction regimen biologically equivalent to a single-dose 23 Gy fraction treatment. For the present study, the total 3D-CRT dose was approximated to 64 Gy, delivered as 32 × 2 Gy fraction scheme (Appendix). Finally, Equation (1) was applied to calculate the equivalent 3D-CRT integral dose both for NTT and for PTV. Tumor integral dose was evaluated considering a standard α/β ratio of 10 Gy while the normalization of non-tumor integral doses were calculated assuming an α/β ratio of 3 Gy. Dose normalization for OARs was performed taking into account most recent literature α/β ratio for different organs. 

The choice of these values was inevitably arbitrary since the α/β concept is a non-stochastic concept referring to cell killing and at present it is not known which α/β value might be related to a stochastic effect as the induction of tumors. 

**Figure 1:**
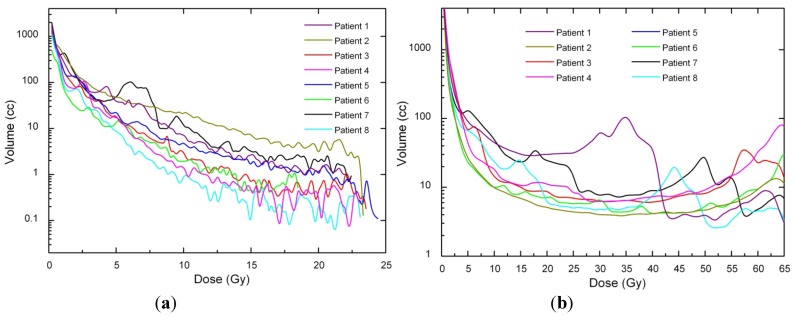
(**a**) Non tumor tissue differential DVH for SBRT. (**b**) Non tumor tissue differential DVH for 3D-CRT.

**Figure 2:**
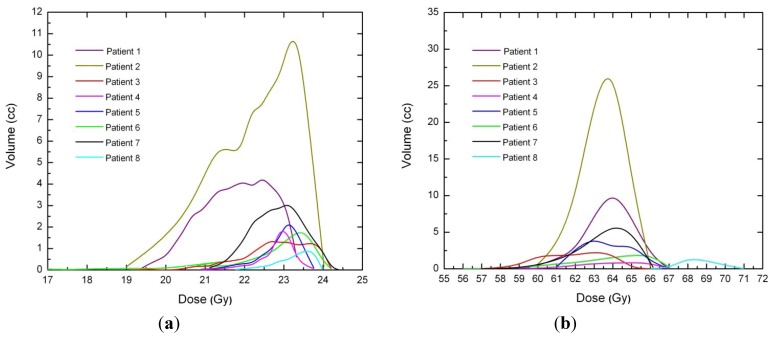
(**a**) PTV differential DVH for SBRT. (**b**) PTV differential DVH for 3D-CRT.

Dose voxels were obtained by differential DVH. For the differential DVH to be calculated, the volumes of interest (PTV, body, OARs) are divided into a volume grid made of equal-sized bins. The doses received by the single volume elements were provided straightforwardly by TPSs. Differential DVHs for NTT and PTVs are reported in Figure 1 and Figure 2 for both techniques.

The maximum treated volume was 86.1 cm^3^ with a median volume of 33.2 cm^3^ (range between 1.5 cm^3^ and 86.1 cm^3^). CT images were acquired with a 4-D CT scanner (LightSpeed^®^ RT and Advantage 4D^®^ respiratory gating) and then registered in order to get a virtual dynamic volume which provided tumour displacements information. In all SBRT plans PTV was obtained expanding GTV (CTV = GTV) with a margin of 5–10 mm. CT slice thickness was 2.5 mm in all patients. For both techniques, OARs, PTV and Body minus PTV were then contoured by an experienced radiation oncologist. The structure “Body minus PTV” was used to calculate the overall non-tumour integral dose (NTID). All the structures were contoured in such a way as not to overlap with adjacent structures (*i.e*., every voxel was assigned to only one structure).

As for SBRT treatment plans, two to five monoisocentic non-coplanar arcs were used. In one case a treatment with eight non-coplanar fixed fields was planned. For all plans, prescription was 90% of the total dose (23 Gy). On the basis of the immobilization equipment, linear margins between BrainLab’s dynamic micromultileaf collimator and PTV were chosen to be 2 mm.

The same structures were then used for 3D-CRT treatment planning (TPS) with Eclipse^®^ software (Eclipse 7.3.10 Varian, Palo Alto, CA, USA). A different number of coplanar fields were used depending upon the tumor localization. All treatment plans were performed for a 6 MV Varian DBX Linac. Linear margins between multileaf collimator and PTV was 5 mm in order to have adequate target coverage. Prescription dose ranged between 94% and 96% of the therapeutic dose (edge of the PTV encompassed by 89–91% isodose curve) and all plans were optimized in order to have mean target coverage at least 95% of the prescription dose.

As for the target, the same setup uncertainties for SBRT planning were considered for 3D-CRT. No margins were added for accounting ITV (Internal Target Volume) since the tumour volume was determined from 4D thoracic CT images, thus accounting for respiratory motion. CTV was obtained expanding GTV with a margin of 0.6–0.8 cm and PTV by a further expansion of 0.5–1.0 cm. A comparison between SBRT and 3D-CRT treatment plans is reported in Table 2.

**Table 2:** Geometrical features and fractionation schemes of SBRT and 3D-CRT plans generated with TPSs.

	SBRT	3D-CRT
Margins GTV → CTV	none	0.6–0.8 cm
Margins CTV → PTV	0.5–1.0 cm	0.5–1.5 cm
Distance collimator-PTV	2 mm	5 mm
Prescription dose	23 Gy × 1 fr to 90% isodose line	2 Gy × 32 fr to 94–96% isodose line
Technique	2–5 noncoplanar arcs or 8 fixed fields	3–4 coplanar fields
Calculation algorithm	Pencilbeam	Pencilbeam
Collimator	microMLC	MLC
Linac Voltage	6 MV	6 MV

All plans were generated with commercially available treatment planning systems (TPS). 3D-CRT dose calculations were performed with Eclipse^®^ implemented with pencil beam convolution algorithm and with BATHO methods for the inhomogeneity corrections. All SBRT plans were generated with BrainSCAN TPS (BrainSCAN^TM^ v.5.2.1, BrainLAB AG. Heimstetten, Germany) implemented with pencil beam algorithm and heterogeneity corrections as well.

All patients were treated with a 6 MV Varian DBX Linac since voltages below 6 MV are always recommended when irradiating tumors surrounded by lung because of the smaller penumbra widening. This recommendation is also suggested by the smaller difference found between the experimental and the predicted percentage depth doses (PDDs) inside the lung, when correction-based algorithms are used [32].

The dosimetric characteristics of both linear accelerators were measured during acceptance testing and commissioning and their consistency with dose calculated by respective TPSs were verified. Measurements have shown excellent agreement between dose delivered and that calculated by both TPSs, with absolute dose difference being consistently within 1.0% for Eclipse and within 0.9% for BrainLAB TPS. Analyses were performed by using a paired two-tailed Student *t*-test to determine if there was a significant difference (*p* ≤ 0.05) between the data.

## 3. Results and Discussion

As a result of the hypofractionated dose delivery scheme and the higher sensitivity to fractionation of late-responding tissues, in all SBRT plans the NTID increased compared to 3D-CRT plans (*p* = 0.002) (Table 3) while, as expected, no significant difference of ID to PTVs were observed between the techniques (Table 4), with *p* = 1. In fact, both for 3D-CRT and SBRT, the lesions were planned with the intent of providing the same target coverage and maintaining approximately the same tumor average dose.

**Table 3:** Non-Tumour Integral Dose (Gy × liter) and increase percentage of SBRT respect to 3D-CRT. Abbreviations: ID = integral dose; 3D-CRT = three-dimensional conformal radiotherapy; SBRT = stereotactic body radiation therapy; EQID = integral dose normalized. Statistically significant difference were found (*p* = 0.002).

Cases	NTT Volume	3D-CRT	SBRT	ID 3D-CRT	SBRT EQID
	(liters)	Technique	Technique	(2 Gy × 32)	(23 Gy × 1, α/β = 3Gy)
Case 1	29.1	3 fixed fields	8 fixed fields	59.2	88.7 (+49.8%)
Case 2	23.4	2 fixed fields	2 arcs	67.9	123.6 (+82%)
Case 3	35.3	4 fixed fields	2 arcs	31.8	51.5 (+61.9%)
Case 4	23.1	3 fixed fields	3 arcs	20.8	38.6 (+86%)
Case 5	25.1	3 fixed fields	4 arcs	40.2	78.0 (+83%)
Case 6	20.5	3 fixed fields	4 arcs	18.5	51.5 (+178%)
Case 7	30.8	3 fixed fields	4 arcs	33.9	111.3 (+228%)
Case 8	20.5	3 fixed fields	5 arcs	18.5	33.6 (+81%)

**Table 4:** PTVs integral dose (Gy × liter). As expected, no significant difference of ID to PTVs were observed (*p* = 1).

Cases	PTV	ID 3D-CRT	SBRT EQID
	Volume (cl)	(2 Gy × 32, α/β = 10 Gy)	(23 Gy × 1, α/β = 10 Gy)
Case 1	47	3.02	2.90
Case 2	86.1	8.21	8.58
Case 3	12.5	0.86	0.76
Case 4	3.9	0.25	0.25
Case 5	14.4	0.86	0.88
Case 6	8.8	0.56	0.40
Case 7	23.7	1.52	1.50
Case 8	1.5	0.10	0.11

**Figure 3:**
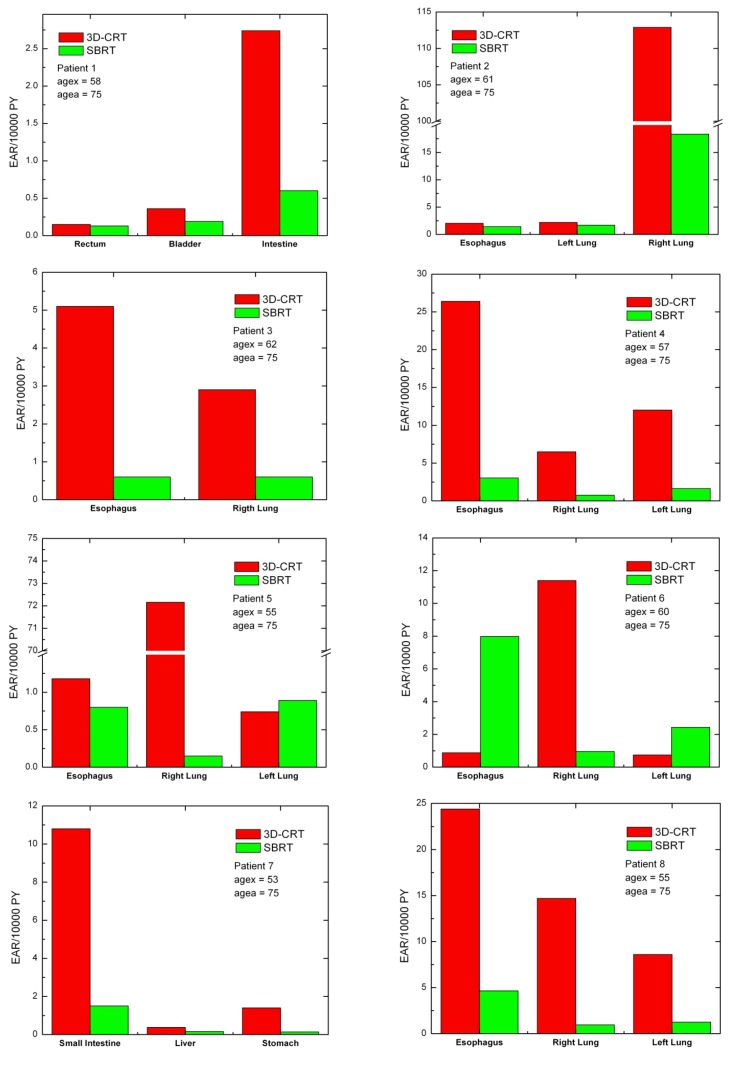
Excess absolute cancer risk for each patient, for the OARs. EARs were calculated from DVHs according to Equation (1).

For each patient, EARs for the main organs of interest were calculated (Figure 3). As a general rule, EARs for SBRT patients are smaller than secondary cancer risk for patients that received standard conformal radiation therapy. Exceptions are represented by patients 5 and 6, where EARs for left lung (patients 5 and 6) and esophagus (patient 6) are higher for SBRT rather than 3D-CRT.

**Figure 4:**
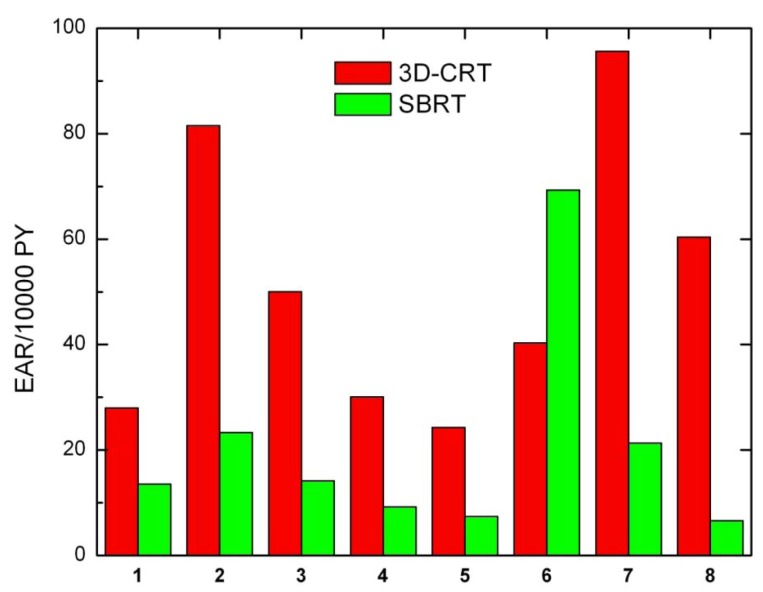
Excess absolute cancer risk for all solid tumors, for all patients.

Secondary cancer risk for all solid tumors was also calculated for each patient (Figure 4). Except for patient 6, EARs for SBRT are systematically lower than EARs obtained for 3D-CRT.

As a general rule, non tumor integral dose depends on a number of factors. As reported by D’Souza [13] beam margin size and beam energy are the most relevant parameters, with smaller margin and higher energy consistently reducing NTID regardless of the number of beams. In the present study, given the same beam energy (6 MV), one would expect that the smaller margin used in SBRT (2 mm *vs*. 5 mm used in 3D-CRT) lead to a reduction of the NTID. Actually, the hypofractionated dose delivery scheme used in SBRT increases the (normalized) average dose to non tumor tissues, thus increasing the NTID as well; in fact, according to the dose normalization rule, low α/β ratio are more sensitive to higher dose per fraction. According to the same work, number of beams, beam direction and relative beam weight have little effect on NTID.

When evaluating radiation dose to healthy tissues, especially at sites remote from the treatment region, radiation leakage may represent an important factor, increasing integral dose to normal structures. Specifically, two main sources of leakage can be considered to increase the patient NTID: transmission of radiation through the collimator leaves and leakage through the primary collimation system. It is generally believed that leakage through the leaves of a conventional collimator might carry a significant contribution to peripheral integral dose (leakage is about 2.5% for 6 MV photon beams).

More specifically, leakage radiation might play a crucial role in some special techniques. A recent work by Petti *et al*. [33] showed that peripheral dose in CyberKnife radiosurgery is due largely to leakage radiation. They measured the radiation dose at different distances outside the treatment field finding that for distances larger than 40 cm from the field edge radiation leakage is the dominant component and is directly related to the number of MU delivered. Dose values due to leakage radiation resulted two to five times higher than those measured for the comparable gamma knife brain treatment, and up to a factor of four times larger those measured in the IMRT experiment.

Over the last decade integral dose has aroused a lively interest due to its alleged correlation with secondary cancers. Radiation-induced secondary malignancies are rare, but since treatment techniques improve and clinical outcomes are improving accordingly, secondary tumor risk after oncologic treatment might represent a relevant issue. Of course, the risk of secondary cancer induction from radiation treatments is likely not to be worrisome within a few years after treatment given the latency period of malignancies, but radiation-induced secondary cancer might be a relevant concern for those (especially young) patients whose progression free survival is greater than 5 years. Different works show that disease-free survival is rapidly increasing for patients which undergo SBRT; Uematsu and *et al*. [34] reported 5-year results of treatment of 50 patients with stage I NSCL finding a 3-year survival rate being as high as 66%, while Nagata et colleagues [35] found overall survival rates after 3 years for stage IA and IB lung cancer being 83% and 72%, respectively.

In the present paper we used a dose-response relationship for cancer induction that includes fractionation effects, therefore suitable for radiotherapy applications. Under the assumptions made and according to the model used our study indicates that despite the fact that for all patients integral dose is higher for SBRT treatments than 3D-CRT, secondary cancer risk associated to SBRT patients is significantly smaller than that calculated for 3D-CRT. This suggests that integral dose may not be a good estimator for quantifying cancer induction. This is imputable to the fact that in the case of SBRT the dose-response curve for carcinoma induction is highly non-linear. In the present study we assumed a bell-shaped behavior, consequently leading to lower cancer induction rates. Furthermore, integral dose does not consider fractionation at all, and fractionation is supposed to be directly correlated to an increased cancer risk [31]. Our findings are in agreement with a recent study by Schneider and colleagues [31] showing that hypofractionated radiotherapy has the potential for secondary cancer reduction.

It is worth noting that the results presented in this study are valid under the assumptions made: neither neutron dose nor leakage radiation accounted and risk calculations performed on the plane of interest for the treatment. In the present analysis a limitation of DVH computation might be represented by the use of Pencil Beam algorithm, which is known to have some drawbacks in low density media. Nevertheless, possible dosimetric inaccuracies are likely to affect DVH calculations (and the following DVH-related evaluations) in both TPS in the same magnitude. Despite the use of more advanced dose evaluations algorithm is encouraged for lung treatments (anisotropic analytical algorithm and collapsed cone convolution), pencil beam algorithm is still widely used and implemented in the clinical practice both for standard 3D-CRT and SBRT of lung malignancies [36,37]. In such case, voltages below 6 MV are always recommended and dose evaluations with heterogeneity corrections are necessary [38,39].

Finally, some consideration about the application of the linear-quadratic (LQ) model are needed. In fact, in radiotherapeutic applications, the LQ formalism is the tool most commonly used for quantitative predictions of dose/fractionations dependencies. Questions may arise when using the LQ model to describe dose response in the high dose per fraction range. 

At present, the LQ model is reasonably well validated (experimentally and theoretically) and its use is reasonable up to several Grays per fraction. In addition, there is a fairly wide range of studies for which it is possible to test concordance with the LQ predictions in the 2 to 20 Gy range [35]. Different works show that different quantitative in vivo endpoints are consistent with LQ model over a wide range of doses per fraction, including those of interest in hypofractionation [40].

At higher doses the LQ model might have deficiencies and experimental survival curves suggest a purely linear rather than a continuation of the linear-quadratic shape (continuously bending) so LQ model may not properly evaluate the tumor dose, predicting more cell kill.

Caution is advised in the presence of bio-mathematical model which use radiobiological parameters. In fact, although mathematical models are widely used to compare different radiotherapy technique and different fractionation scheme, the radiobiological parameters on which they are funded are still not completely optimized, thus unavoidably introducing some degree of approximation.

## 4. Conclusions

Our results indicate that although NTID was greater in SBRT than in 3D-CRT, secondary cancer risk associated to SBRT patients is significantly smaller than that calculated for 3D-CRT. This suggests that integral dose may not be a good estimator for quantifying cancer induction.

The development of reliable secondary cancer risk models seems to be a key issue in fractionated radiotherapy and comparisons of integral dose received with 3D-CRT and other special techniques are strongly encouraged.

## Appendix

### Linear Quadratic Model and BED Formalism

In the classic Linear Quadratic (LQ) model, the cell fraction surviving an irradiation (*SF*) is described by the following function:

(A-1)

where *d* is the fraction dose (delivered as a single acute fraction), and α and β are tissue specific parameters which characterize the cell intrinsic radiosensitivity (the constant α is the log_e_ cell kill per Gy of the initial linear component and β is the loge cell kill per Gy^2^ of the quadratic component of the survival curve). The α/β ratio represents the relative importance of the linear and quadratic terms. Early reacting tissues such as the skin, the intestinal epithelium, and tumours have a large α/β value of about 10 Gy, whereas late reacting tissues such as the brain and bone have a smaller α/β value of 2–3 Gy.

Given *n* fractions of the same dose *d*, the total surviving fraction *SF_tot_* is given by the product of the single surviving fractions (multiplication rule for independent events):

(A-2)

Thus the total biological effect *E* can be expressed as:

(A-3)

Biological Effective Dose (*BED*) is defined as the biological effect *E* divided by α:

(A-4)

*BED* has the dimensions of dose, and represents a quantity by which different fractionation regimens can be compared. In fact, two fractionation schedules are taken as “equivalent” if they give the same radiobiological effect. Two irradiation schedules with different fractionation (*n_1_d_1_* and *n_2_d_2_*) can be compared by equating the *BED*s for the two regimens:

(A-5)

Equation (A-5) was used to calculate the equivalent tumour dose for a standard fractionation regimen (3D-CRT), with *d_2_* = 2Gy, which is biologically equivalent to a single-dose (*n_1_* = 1) fraction SBRT treatment with *d_1_* = 23Gy. If the tumour *BED* between 3D-CRT and SBRT is to be maintained constant, considering an α/β = 10Gy, Equation (A-6) becomes:

(A-6)

For the present study, *D_3D-CRT_* was approximated to 64 Gy, delivered as 32 × 2 Gy fraction scheme.
